# NaCl pellets for prospective dosimetry using optically stimulated luminescence: Signal integrity and long-term versus short-term exposure

**DOI:** 10.1007/s00411-020-00873-8

**Published:** 2020-09-23

**Authors:** Lovisa Waldner, Christopher Rääf, Christian Bernhardsson

**Affiliations:** Department of Translational Medicine, Medical Radiation Physics, Lund University, Malmö, Sweden

**Keywords:** OSL, Dosimetry, Salt, Fading, Extended exposures, Preheat

## Abstract

Optically stimulated luminescence (OSL) signal properties of pellets from three types of NaCl (two household salts and one analytical grade salt) were investigated for their use in prospective dosimetry. Special attention was given to the OSL signal behaviour with time. The readout protocol was optimised in terms of preheat temperature, and the OSL signal yield of the NaCl pellet with time as well as the fading of the OSL signal with time was investigated. The effects of acute and chronic irradiations were compared. Irradiations and readout were performed using a Risø TL/OSL reader (TL/OSL-DA-15, DTU Nutech, Denmark). The optimal preheat temperature was determined to be 100 ºC, yielding OSL signals similar to a 1 h pause before OSL signal readout. There was no OSL signal fading observed as a function of time, but a decrease in the OSL signal yield of the NaCl pellets with time resulted in an apparent inverse fading when converting the OSL signal to an absorbed dose. For chronic radiation exposures of up to five weeks, the sensitivity of the NaCl pellets was found to be stable. The results of this study show that the use of NaCl pellets for prospective dosimetry is a promising, cost-effective, and accessible complement to commercially available alternatives for accurate absorbed dose determinations.

## Introduction

Prior studies on optically stimulated luminescence (OSL) dosimetry involving luminescent materials have shown that ordinary household salt (NaCl) has potential applications both in retrospective dosimetry (Bailey et al. 2000; Bernhardsson et al. 2009; Spooner et al. 2011; Hunter et al. 2012; Ademola 2017) and prospective dosimetry (Bernhardsson et al. 2012; Ekendahl et al. 2016; Christiansson et al. 2018; Waldner and Bernhardsson 2018; Majgier et al. 2019). As NaCl is easily accessible all over the world, it could be an available and cost-effective complement to commercially available dosimeters in applications where a large number of dosimeters is needed or when there is a shortage of other available means for in situ or individual dosimetry. Such applications may include personal dosimetry and radiation dose assessments in hospitals, the nuclear industry, or research environments; environmental monitoring; and emergency preparedness. Compressing the NaCl into pellets simplifies handling, improves reproducibility, and further supports the potential use of NaCl for prospective OSL dosimetry. Some of the dosimetric properties of NaCl in pellet form have already been investigated (Elashmawy 2018; Waldner and Bernhardsson 2018; Majgier et al. 2019), but the use of NaCl pellets for long-term measurements outside the laboratory, e.g*.*, for personal dosimetry or environmental monitoring, requires more knowledge and understanding regarding any potential changes in signal properties over time. Until now, most of the studies on NaCl pellets have only included results from acute radiation exposures, e.g., inside the reader. However, under realistic conditions, the dosimeter may be continuously exposed over days or weeks. It is not yet known if and how such chronic exposures affect the radiation-induced OSL signal used for the quantification of absorbed doses in NaCl. Furthermore, the effect on the OSL signal due to compressing the NaCl to pellets have not yet been fully investigated, but initial investigations (Waldner 2017) indicate that the dosimetric properties are affected when grains of NaCl are compressed to pellets, yielding higher weight-normalised signals for pellets as compared to grains of salt.

A common practise in OSL for retrospective dosimetry and archaeological dating is to apply a preheat before readout (Bailey 2000; Christiansson et al. 2011); a pause between irradiation and readout, to let the signal stabilise on its own, has also been suggested (Waldner and Bernhardsson 2018). Multiple measures are needed to achieve a stable signal depending on the available readout equipment and labour time. Consequently, further studies are needed to find an optimised preheat temperature for the NaCl pellets and to determine how to achieve a stable and reproducible OSL signal at the readout.

The overall aim of this work was to investigate the dosimetric properties of NaCl pellets over time, both during and after exposure. More specifically, the aim was to investigate the potential OSL signal fading, the OSL signal yield of the NaCl pellets over time, and the effect of chronic vs acute exposures, and to optimise the preheat temperature. Knowledge on these issues is essential for dose applications outside the laboratory where complex measurement conditions may dominate, and when handling a large number of NaCl pellets.

## Method

### Production of NaCl pellets

The study examined three different salts (NaCl) (Table 1) compressed to pellets (Waldner and Bernhardsson 2018). Salt 1 was a commercially available rock salt while Salt 2 was a commercially available sea salt, both bought in Swedish supermarkets. Salt 3 was an analytical grade salt used for laboratory purposes (Scharlab, 99.5%). The NaCl pellets were produced using a specially-made tool (Promech, Sweden) and a hydraulic hand press (Hamron, Sweden) with a pressure of 0.8 tons per pellet. The pellets were 4 mm in diameter and about 0.8 mm thick, made from about 20 mg of NaCl with grain sizes between 100 and 400 µm. The pellets were produced at least 24 h before they were exposed to ionising radiation unless an exception is noted for the different experiments. After manufacturing the pellets were kept at room temperature in a laboratory with a combination of natural and artificial lighting. No additional optical or thermal bleaching was needed to zero potential background OSL signals. The produced pellets were visually identical and no additional controls were made to ensure that they were physically and dosimetrically identical beyond the properties previously investigated in Waldner and Bernhardsson (2018). In addition, any difference in dosimetric properties between pellets was corrected by the use of the calibration dose.

**Table 1:** Three types of salt used in the current study: Salts 1 and 2 were ordinary table salts bought in supermarkets, while Salt 3 was an analytical grade NaCl for laboratory purposes

Salt #	Type of salt	Name, company
1	Rock salt	Falksalt Finkornigt hushållssalt, Hanson and Möhring, Sweden
2	Sea salt	Falksalt finkornigt medelhavssalt, Hanson and Möhring, Sweden
3	Analytical, reagent grade salt	Scharlau, Scharlab, Spain

### OSL signal readout

Readout of the OSL signal from NaCl pellets was performed using a Risø TL/OSL reader (TL/OSL-DA-15, DTU Nutech, Denmark), described in detail by Thomsen (2004). The reader was equipped with an internal ^90^Sr/^90^Y irradiation source (20 MBq as of 9 April 2009) with an absorbed dose rate of 0.77 ± 0.02 mGy s^−1^ (as of 1 February 2019) to quartz (calibration quartz, DTU Nutech, Batch 101). The dose rate to NaCl was 0.72 mGy s^−1^, calculated using a stopping power ratio of 0.938 (Berger et al. 2005) between NaCl and SiO_2_. Light stimulation was performed using continuous wave (CW) stimulation by means of blue (λ = 470 nm) LEDs. The photomultiplier tube used to detect the luminescence photons was equipped with a 7.5 mm thick Hoya U-340 filter, resulting in a detection window with maximum transmission at 340 nm.

Typically, the first minutes after irradiation the OSL signal decreases rapidly before it starts to stabilise after about 1 h (Waldner and Bernhardsson 2018), due to the emptying of the more shallow and unstable impurity traps in the NaCl. It is desirable to avoid any signal contributions of these shallow traps, which means that the unstable traps need to be emptied before readout of the OSL signal. This can be achieved by either waiting for the signal to stabilise on its own at room temperature or by heating the sample to remove the unstable part of the signal.

To investigate the optimal use of either a preheat or a pause, the readout protocol in Table 2 was used for OSL signal readout. The preheat temperature was kept the same in Steps 2.1 and 5.1 in Table 2 to ensure a reproducible fraction of emptied energy state traps in the crystal lattice after each irradiation.

**Table 2:** The protocol for OSL-signal readouts of NaCl pellets using the Risø TL/OSL reader

Step	Operation and readout settings
1	Administration of the unknown dose, *D*_*u*_, as accumulated during exposure or using the internal ^90^Sr/^90^Y source
2.1	Preheat to a set temperature, *T*, at a heating rate of 5 ºC/s and hold at *T* for 10 s
2.2	Pause for 1 h
3	Readout of *S*_*u*_ at ambient temperature, continuous OSL at 40% (~ 16 mW cm^−2^) of maximum blue (λ = 470 ± 30 nm) LED intensity during 10 s
4	Administration of a calibration dose, *D*_*c*_, using the internal ^90^Sr/^90^Y source
5.1	Preheat to set temperature, *T*, at a heating rate of 5 ºC/s and hold at *T* for 10 s
5.2	Pause for 1 h
6	Readout of *S*_*c*_ at ambient temperature, OSL at 40% of maximum blue LED intensity for 10 s

Either Steps 2.1 and 5.1 or Steps 2.2 and 5.2 were used for readout depending if a preheat or a pause was used. *S*_*u*_ signal after exposure to unknown dose; *S*_*c*_ signal after exposure to calibration dose

Pellets of the three types of salt presented in Table 1 were subjected to the two readout protocols described in Table 2. In the assessment of the unknown signal, *S*_*u*_, no background correction was performed as the background signal was assumed negligible (all NaCl pellets had been pre-bleached in daylight). After the 1 h pause or preheat after exposure, the radiation-induced signal from each pellet was defined as the integrated number of luminescence counts registered during the first 5 s of the 20-s OSL signal readout with blue light stimulation. The OSL signal decreased rapidly during the first seconds of readout to a background which then decreased slowly. The integrated luminescence during the last 5 s of the recorded OSL signal decay curve was used as a background correction when further irradiations and readouts were performed on the same pellet since any additional induced signal will be added to the remaining background from the former readout. The calibration signal, *S*_*c*_, was thus defined as the integrated OSL signal between 0 and 5 s of readout corrected for the background of the previous OSL signal readout between 15 and 20 s.

For some experiments, only Steps 1–3 of the readout protocol in Table 2 were used, and the signal was compared to the pre-established signal-dose response curve described in Waldner and Bernhardsson (2018). This curve shows the mean OSL signal from 10 NaCl pellets, read using Steps 1–3 of the protocol in Table 2, for several different increasing absorbed doses up to 300 mGy. Using such a curve provides a quick estimation of the absorbed dose, with uncertainties of about 10% (Waldner and Bernhardsson 2018) in the absorbed dose determination.

The procedure of using only the readout Steps 1–3 in Table 2 was also used to determine the calibration dose when the full readout protocol in Table 2 was used (Section “OSL signal fading and its dependence on preheating conditions”). According to previous work (Waldner and Bernhardsson 2018), a *D*_*c*_ twice the size of *D*_*u*_ gives a good estimation of the absorbed dose for all salts at the absorbed dose levels used in the present investigation (< 50 mGy).

When using the full protocol in Table 2, the absorbed dose to the NaCl pellets was calculated according to Eq. 1.

1 $${D}_{u}=\frac{{S}_{u}}{{S}_{c}}\cdot {D}_{c},$$

where *D*_*u*_ is the unknown dose, *D*_*c*_ is the calibration dose, *S*_*u*_ is the signal after exposure to an unknown dose, and *S*_*c*_ is the calibration signal.

### Optimisation of preheat temperature for NaCl pellets

The preheat temperature was optimised in two steps: first, it was determined whether heating the NaCl pellet affected its dosimetric properties, and second, it was investigated whether the chosen temperature was sufficient to empty the unstable electron traps in the crystal lattice.

#### Effects of preheating the NaCl pellet

When using a calibration dose to determine an unknown dose, it is important that the OSL signal per unit dose is the same for both the unknown exposure and the administered calibration dose (or that the relationship between the responses is known and constant). If the dosimetric properties change between the two irradiations, the unknown dose, *D*_*u*_ in Eq. 1, will not be estimated correctly.

To investigate if the heating of NaCl before readout affects the dosimetric properties, and if so, at what temperature, several measurements were performed using the protocol in Table 2. The preheat was varied between 25 and 225 ºC in steps of 25 ºC, with all other parameters remained unchanged. The preheat temperature in Step 2.1 was kept the same as that in Step 5.1 (Table 2). For this investigation, the two given doses, *D*_*u*_ and *D*_*c*_, had the same value (21.6 mGy); hence, the corresponding signals *S*_*u*_ and *S*_*c*_ were expected to be similar. The ratio of the unknown signal *S*_*u*_ and the calibration signal *S*_*c*_ was calculated and compared for the different preheat temperatures. Any deviation from a value of 1 for this ratio was then interpreted as a change in sensitivity.

#### Time delay between preheat and readout

To investigate if the selected preheat was sufficient to empty the unstable traps in the NaCl, the OSL signal was read at several instances after the preheat. If the signal did not continue to decrease after preheat, then the chosen preheat temperature was considered to be sufficient. Five NaCl pellets were irradiated with an absorbed dose of 21.6 mGy and then preheated. After a pause following the preheat, the OSL signal was read. This was repeated nine times, with pauses between 0 and 400 s introduced between the preheat and readout steps, i.e., between Steps 2.1 and 3 in the readout protocol in Table 2.

### OSL signal fading and its dependence on preheating conditions

In the present study, fading is defined as the decrease in the read OSL signal, *S*_*u*_, and estimated dose, *D*_*u*_, over time. To investigate the fading, the OSL signal was read on multiple occasions from different pellets after exposure to the same absorbed dose. For this, 100 NaCl pellets were irradiated by a ^60^Co gamma radiation source (Gammatron 3, Siemens, Germany) 24 h after production. The NaCl pellets were positioned in a PMMA phantom with a 5 mm build-up layer and then irradiated with an absorbed dose, *D*_*u*_, corresponding to 5 mGy to water. After this exposure, the pellets were kept in a light sealed plastic container intended for photographic films, to keep the signal from optical bleaching.

The OSL signal from the irradiated pellets was read at various times, from 1 h to 4 weeks after irradiation. Ten pellets were read on each occasion: five at room temperature after being preheated to the temperature determined in Section “Production of NaCl pellets” and five at room temperature without any preheat. After readout, the signal was compared to a pre-established signal-dose calibration curve (Waldner and Bernhardsson 2018) to estimate the absorbed dose from the ^60^Co source. Based on the estimated absorbed dose, a calibration dose, *D*_*c*_, was determined and administrated to the pellets. For all three salts, *D*_*c*_ was set twice as high as the administered dose, *D*_*u*_. The calibration signal, *S*_*c*_, was read after a preheat of 100 ºC for the first five pellets and after a 1-h pause for the other five pellets. The estimated absorbed dose was calculated using Eq. 1.

### OSL signal yield over time

To investigate the OSL signal yield over time, NaCl pellets were irradiated, and the OSL signal read, on several occasions between one hour and one month after production of the pellets. The internal ^90^Sr/^90^Y source of the TL/OSL reader was used for the irradiations. After the pellets were manufactured they were kept in a transparent plastic container at ambient conditions. The *D*_*u*_ administered to the NaCl pellets was at the same level (28.8 mGy) for each exposure. Two types of readout (Table 2) were used after irradiation: first, five pellets were read after a preheat (Step 2.1 in Table 2) at a temperature determined in Section “Production of NaCl pellets”, and second, five pellets were read after a pause of one hour using no preheat (Step 2.2 in Table 2). In this way, the OSL signal yield, or radiation sensitivity, of the NaCl pellets was determined as a function of time after pellet production for two types of signal acquisition.

### Acute versus chronic irradiation

**Fig. 1:**
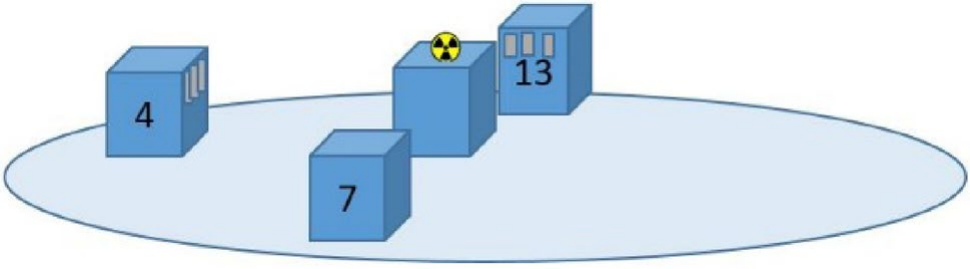
The setup as viewed from the side for prolonged irradiation using a ^137^Cs source of 20 MBq. NaCl pellets were placed on styrofoam blocks (blue) at three different distances from the source to achieve three different dose rates, indicated on the blocks in units of µSv/h (ambient dose equivalent as measured by a handheld detector), in a “free air” exposure geometry. The NaCl packages are indicated on the styrofoam blocks in the picture by the small grey boxes

To investigate any potential effects of long-term irradiation and the influence of exposure with varying dose rates, NaCl pellets made from Salt 1 were placed in opaque packages on styrofoam blocks at different distances (40, 60 and 80 cm) from a 20 MBq ^137^Cs point source (Fig. 1). The use of varying distances from the point source was intended to mimic free air exposure with different ambient dose equivalent rates 4, 7, and 13 µSv/h, respectively, as determined using a handheld radiation detector (identiFINDER, FLIR, United States). These dose rates resulted in cumulated doses of around 3–10 mGy to the NaCl pellets after 5 weeks which is well above the detection limits of the NaCl pellets but in the relevant range for personal dosimetry. At each distance from the radiation source, five packages of pellets, each containing five NaCl pellets, were placed on individual styrofoam blocks. One package per distance was removed for readout (full readout protocol with pause, Table 2) after 1, 2, 3, 4, and 5 weeks of exposure to achieve five different total absorbed doses with three different dose rates. The dose rate effects were expected to be the same for the three salts (Table 1), which is why only one salt (Salt 1) was investigated. Scattering effects were not considered as the aim was only to have a constant dose rate at each styrofoam block for the 5 weeks of measurements.

### Uncertainty calculations

Uncertainties were calculated using error propagation (Eq. 2). When irradiating and reading the OSL signal twice from the same NaCl pellet, i.e., twice repeating Steps 1–3 in Table 2, all dosimetric properties were assumed to be unchanged. This means that the unknown signal and calibration signal were not independent of each other but were likely correlated. Thus, their covariance needed to be accounted for in the uncertainty estimate.

2 $${\left(\frac{{\sigma D}_{NaCl}}{{D}_{NaCl}}\right)}^{2}=\left({\left(\frac{\sigma {S}_{u}}{{S}_{u}}\right)}^{2}+{\left(\frac{\sigma {S}_{c}}{{S}_{c}}\right)}^{2}+{\left(\frac{\sigma {D}_{c}}{{D}_{c}}\right)}^{2}-2\cdot r\cdot \frac{\sigma {S}_{u}}{{S}_{u}}\cdot \frac{\sigma {S}_{c}}{{S}_{c}}\right),$$

where *σ* denotes the uncertainty of the previously described variables and *r* is the correlation factor.

**Fig. 2:**
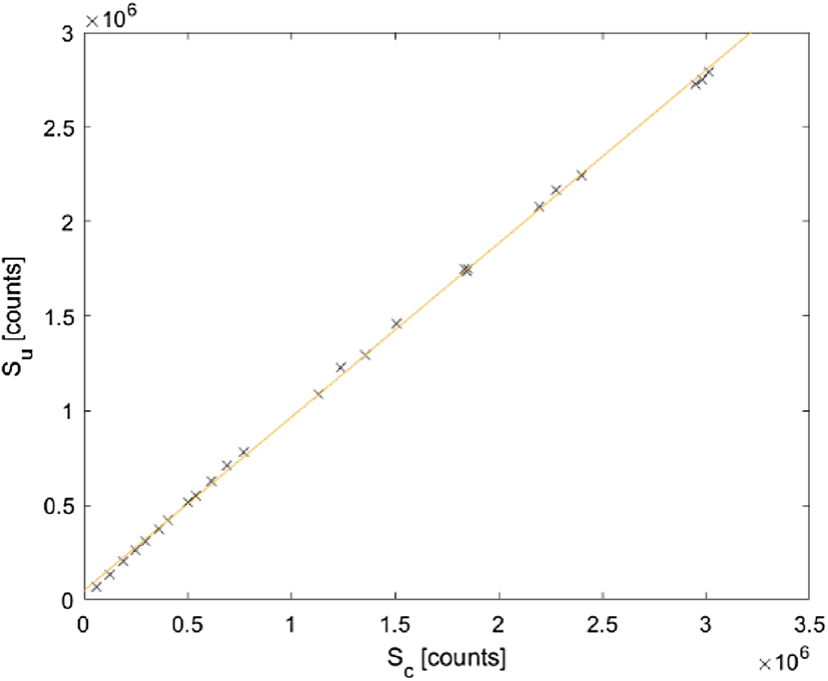
Correlation between the measured signal (*S*_*u*_) and the calibration signal (*S*_*c*_). *R*^2^ was 0.9993 in the graph, giving a correlation, *r*, of 0.9996. The experimental uncertainty, in the order of 2%, is smaller than the symbols used in the graph

Figure 2 shows a graph of *S*_*u*_ as a function of *S*_*c*_, read from the same NaCl pellet after two exposures with the same dose, repeated for different absorbed doses. The correlation, *r*, between *S*_*u*_ and *S*_*c*_ is obtained as the square root of the Pearson *R*^2^ value in Fig. 2. For these data, the correlation was 0.9996, and the uncertainty contribution from *S*_*u*_ and *S*_*c*_ thus became very small.

## Result and discussion

### Optimisation of preheat temperature for NaCl pellets

#### Effects of heating

**Fig. 3:**
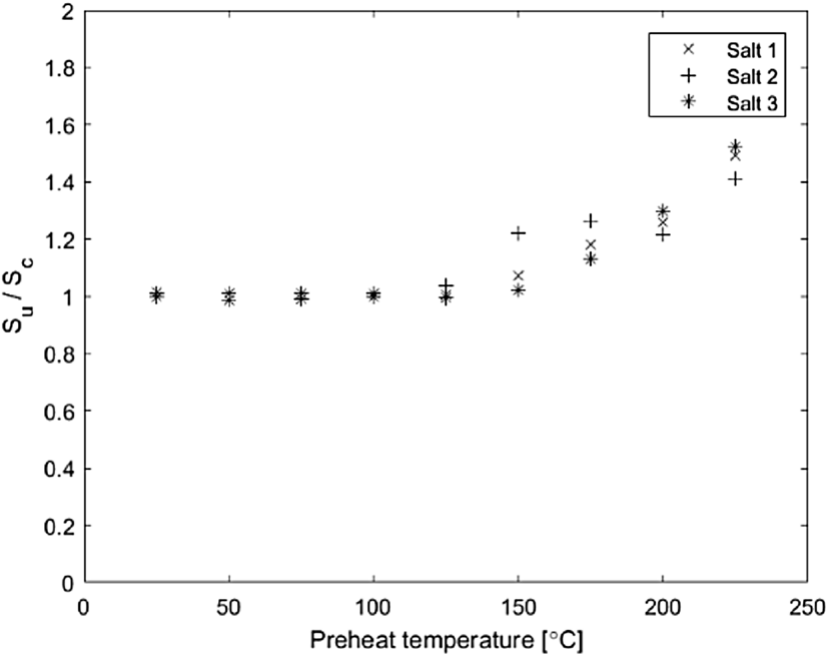
Ratio between the measured blue light CW OSL signal (*S*_*u*_) and the calibration signal (*S*_*c*_) using different preheat temperatures before signal readout. *D*_*u*_ and *D*_*c*_ were fixed at the same value (21.6 mGy). The experimental uncertainties, in the order of 2%, are smaller than the symbols used

In Fig. 3, for the same given dose (*D*_*u*_ = *D*_*c*_), the ratio *S*_*u*_/*S*_*c*_ (see Eq. 1) increases above unity at temperatures above 100 ºC. This means that the calibration signal became lower than the unknown signal if a higher preheat temperature was used. At the time of the first exposure to *D*_*u*_, the pellets had not been heated to temperatures above room temperature. Before the second irradiation, however, the pellets had been preheated to obtain *S*_*u*_. For a correct estimation of the absorbed dose to the NaCl pellets, a preheat must be chosen that does not alter the dosimetric properties of the NaCl.

The aim is thus to preheat the NaCl pellets enough for the unstable energy level traps to be emptied without affecting the properties of the NaCl pellets or emptying the deeper, stable traps in the NaCl crystal. As shown in Fig. 3, 100 ºC is the maximum temperature that can be used for the preheat without altering the dosimetric properties of the NaCl pellets.

It is noted that depending on the heat transfer between the heating element of the OSL reader and the NaCl pellet, the optimal temperature might vary slightly with different readout systems. Furthermore, setting the heating element to 100 ºC might not provide a uniform temperature of the pellet, especially when the heating rate is high and the pellet is only heated for a short period of time.

#### Time delay between preheat and readout

**Fig. 4:**
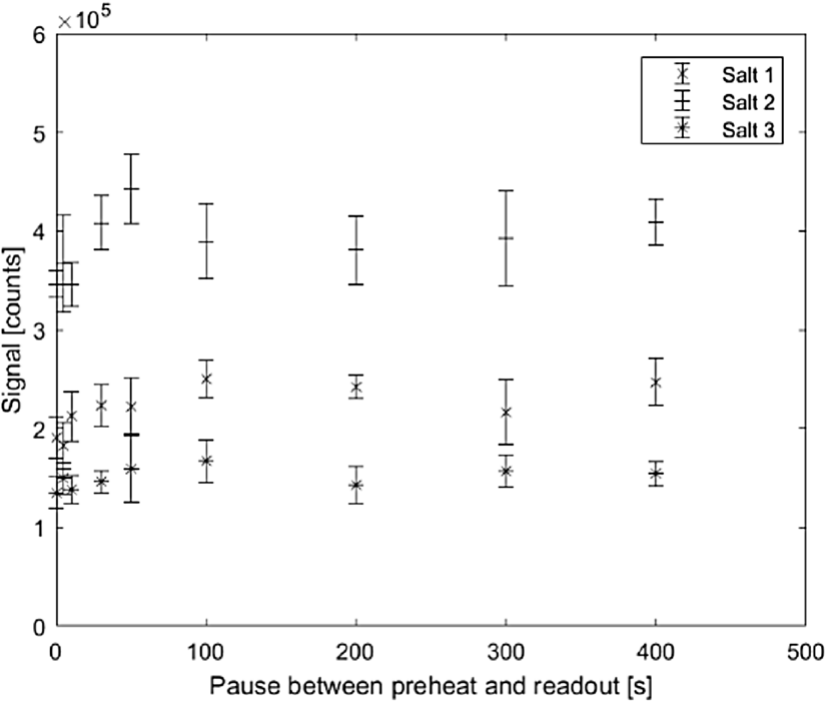
Blue light CW OSL signal in NaCl pellets read at different times after a 100 ºC preheat. The preheat was performed directly after irradiation, after which various pauses (0–400 s) were made before readout. The same absorbed dose was given to all samples (21.6 mGy). Each signal data point represents the arithmetic mean from five pellets, and the uncertainty bars correspond to one standard deviation

To investigate if a preheat of 100 ºC is enough to empty the shallow electron traps, the OSL signal was read at several different occasions after preheat (0–400 s) from different pellets irradiated with the same absorbed dose, 21.6 mGy. The result is shown in Fig. 4. When accounting for the estimated uncertainty (indicated as ± 1 SD uncertainty bars), the signal was observed to be stable after a 100 ºC preheat regardless of the pause time (Fig. 4). This means that the unstable traps were sufficiently emptied and the remaining occupied traps were stable and of importance for absorbed dose estimation. Some variation is to be expected, however, as the OSL signals were compared for different NaCl pellets at the different readout times.

Based on these results, a preheat of 100 ºC was chosen for all further investigations.

### OSL signal fading and its dependence on preheating conditions

**Fig. 5:**
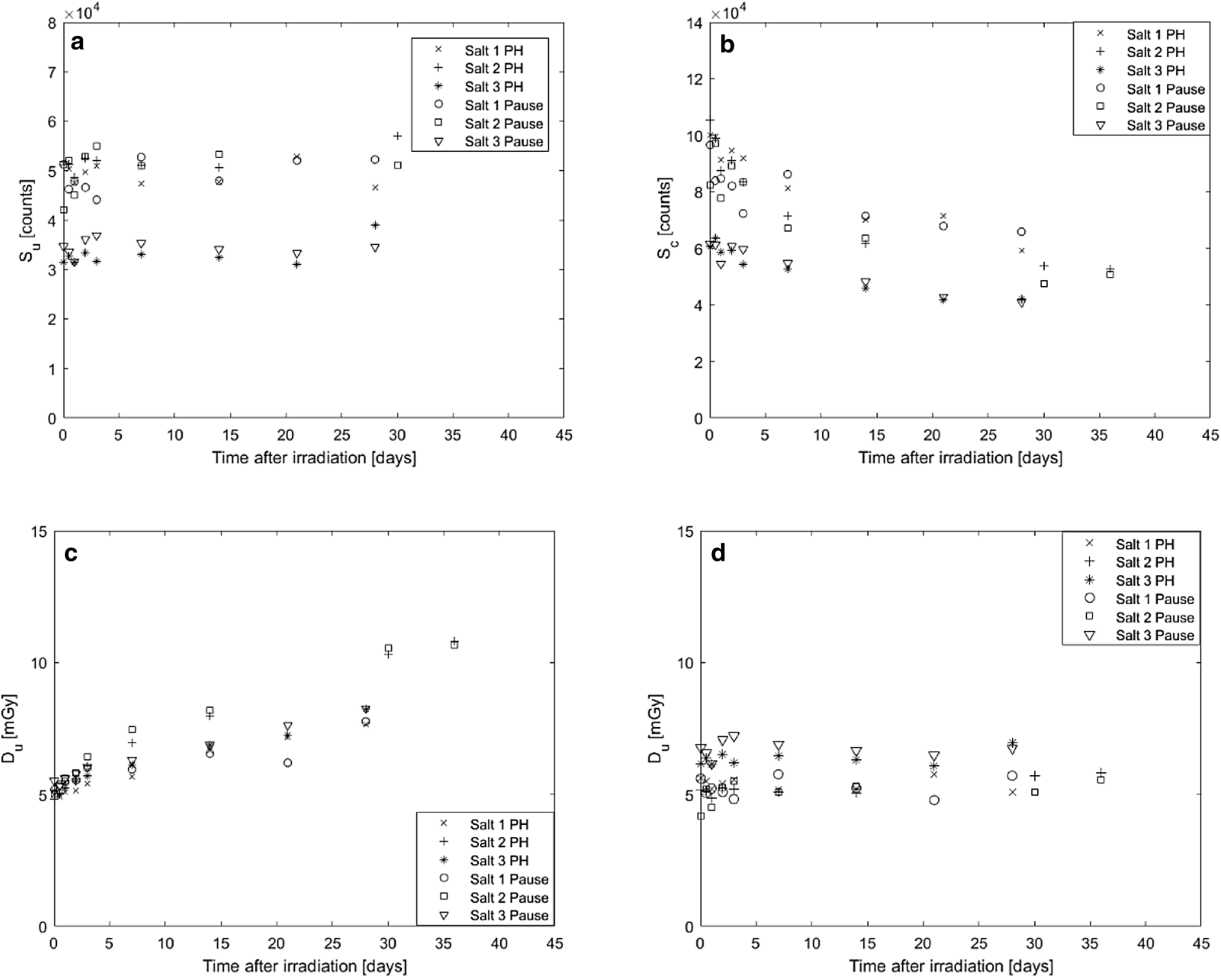
**a** Unknown signal, *S*_*u*_; **b** calibration signal, *S*_*c*_; **c** absorbed doses calculated with calibration dose normalisation; and **d** adsorbed doses calculated with the dose-response curve from (Waldner and Bernhardsson 2018), as a function of time before readout (using either a pause “Pause”, or a preheat protocol “PH” for Salts 1–3. Measurements were performed between 1 h and up to 36 days after irradiation, after a given dose (*D*_*u*_) of 5 mGy. The experimental uncertainties are in the order of 10% for (**a**–**c**) and 2% for (**d**)

Figure 5a–d show (a) the OSL signal over time (non-normalised) when either a pause or a preheat protocol was used; (b) the calibration signal after administration of *D*_*c*_ at different times after the initial *D*_*u*_ irradiation, which gives the OSL signal yield of the NaCl pellets with time; (c) the apparently absorbed dose with time; and (d) the absorbed dose with time when calculated using a predetermined signal-dose response curve (Waldner and Bernhardsson 2018). The rather large variability in Fig. 5a depends on the mass and amount of signal-inducing material of the NaCl pellets. The readout protocol in Table 2 was used, and both readout procedures are presented in Fig. 5.

The three salts show similar trends in all graphs in Fig. 5: the unknown OSL signal, *S*_*u*_, remains stable over 30 days within 1 standard deviation of the uncertainty with no indication of signal fading (Fig. 5a), while the calibration signal decreases with time (Fig. 5b). For all three types of NaCl, this resulted in an apparent inverse fading effect when the absorbed dose, *D*_*u*_, was calculated using Eq. 1 (Fig. 5c). The same trend was obtained and discussed in previous work (Waldner and Bernhardsson 2018). Others have also observed indications of inverse fading of the OSL signal, both for a short time scale of seconds (Biernacka et al. 2016) and a longer time scale of days (Christiansson et al. 2014). However, those observations were related to a different phenomenon than the one observed here. As can be seen in Fig. 5a, there was no fading of the OSL signal itself, and the apparent inverse fading is only attributed to the calibration dose normalisation, *D*_*c*_, in Eq. 1. When dividing by a number which decreases with time, the estimated dose appears to increase with time. However, when *D*_*u*_ is calculated using the signal-dose response curve taken from Waldner and Bernhardsson (2018), the estimated absorbed dose appears stable with time (Fig. 5d). Note that the signal-dose response curve does not correct for any fading, as it was produced by reading the OSL signal 1 h after irradiation. Therefore, the estimated *D*_*u*_ in Fig. 5d will show a similar trend over time as *S*_*u*_ in Fig. 5a, but with an added conversion factor from the signal to the absorbed dose. Since calculating absorbed doses by means of individual calibration doses (Eq. 1) results in more accurate dose estimates than using a pre-established signal-dose response curve, the decreasing signal yield of *S*_*c*_ overtime must be adjusted when using Eq. 1. There are two alternatives to achieve this: either a mathematical correction that is calculated from a graph showing the OSL signal yield with time or avoidance of the use of the pellets until the efficiency is stabilised. However, the first step before applying the second adjustment is to investigate if the OSL signal yield stabilises as a function of time after pellet production.

The OSL signals in Fig. 5a and b were read using two different readout protocols (Table 2), one which used a preheat to empty the unstable traps and one which used a pause. The resulting OSL signals differed in magnitude, but when calculating the absorbed doses (Fig. 5c), the differences cancel and the estimated absorbed dose agreed within about 7% for the two readout protocols. This means that either protocol can be used for absorbed dose estimates with a similar accuracy depending on the available readout possibilities, which in turn may depend on available equipment and time restraints. For example, if only a few samples are to be read, it is faster to use the preheat protocol. However, for a large number of samples, the time difference between a pause or the preheat becomes negligible, and because the heating the samples itself is connected with uncertainties such as heat transfer and uneven heating of the sample (McKeever and Moscovitch 2003), the pause protocol may yield more reliable results.

### OSL signal yield as a function of time

**Fig. 6:**
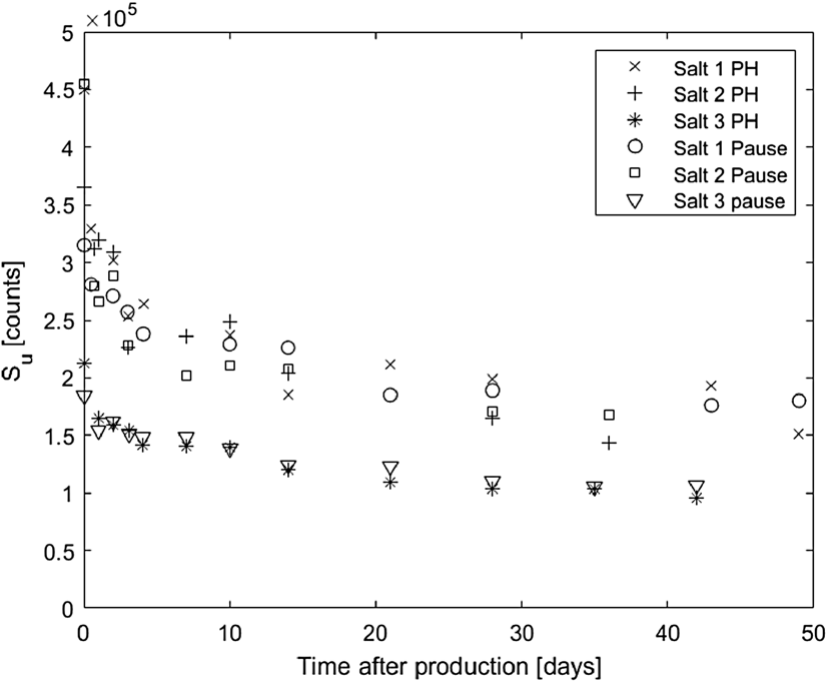
The measured blue light CW OSL signal, *S*_*u*_, after irradiation with an absorbed dose of 28.8 mGy at different times after production of the NaCl pellets (Table 1). The signals were read using the readout protocol in Table 2, with either a pause before readout or a 100 ºC preheat before readout. The uncertainty (± 1 standard deviation) varies between 10 and 20% for each data point

Figure 6 shows the results of exposing NaCl pellets to ionising radiation at different times after production and reading of the OSL signal. It is evident that the signal from the administrated dose, *S*_*u*_, decreases with time even though the administrated dose, *D*_*u*_, was the same for each exposure. This indicates that the NaCl pellets become less sensitive to ionising radiation with time after production. This effect is large during the first two days but tends to stabilise to a certain level of sensitivity after about three weeks after production. If the NaCl pellets are used for prolonged radiation exposure, then it is either necessary to correct for this sensitivity change, or the NaCl pellets need to be produced several weeks in advance before use to allow time for stabilisation of their radiation sensitivity.

The reason for the change in OSL signal yield is not clear, but the compression of the salt during pellet production might be a factor, with point defects becoming instable due to the compression and then starting to stabilise after the stress relaxation. This is further supported by the fact that NaCl pellets generally have a higher weight-normalised OSL signal than grains of salt (Waldner 2017). This needs to be further investigated, however, possibly by performing the same measurements with pellets produced with different compression forces to find a cut-off where the weight-normalised signal from the pellets does not differ from that of the grains, or by investigating the TL curves of the NaCl pellets as a function of time to see if they change in shape over time.

### Correcting for decreasing OSL signal yield

**Fig. 7:**
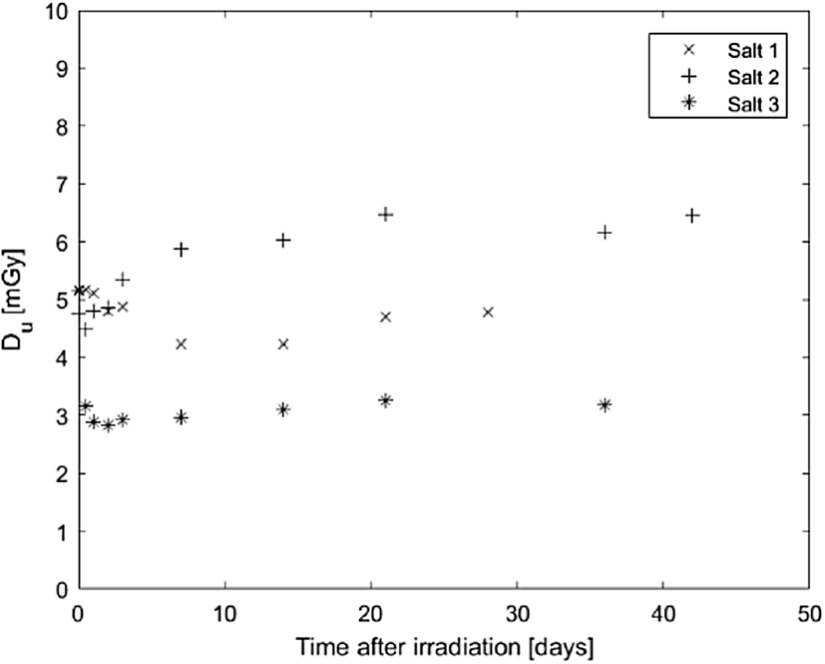
Estimated absorbed dose *D*_*u*_ from Fig. 5c, with corrections for the decrease in efficiency of the OSL signal yield. OSL signals were read using the 3600 s pause protocol. The experimental uncertainties of *D*_*u*_ are in the order of 2%

Using a mathematical correction to obtain a better estimation of the absorbed dose is more time efficient than waiting for the NaCl pellets to age and stabilise. When using Eq. 1 to calculate the absorbed dose, *D*_*c*_ can be adjusted with a time-dependent correction factor that accounts for the time between the first irradiation and the administration of the calibration dose. This correction factor comes from fitting a mathematical function *f*(*t*) to the observed decrease in efficiency with time, *t*. This can be calculated as $f\left({t}_{1}\right)-f\left({t}_{2}\right)$, where *t*_1_ is the time of the first irradiation and *t*_2_ is the time of administration of the calibration dose. Fitting bi-exponential functions to the data of each of the salts in Fig. 6 (pause protocol) and using these to correct the decreasing *S*_*u*_ in Fig. 5b results in the adjusted graphs in Fig. 7. Even after this correction, however, there is still a small increase in the estimated absorbed dose with time, but this increase is significantly smaller compared to that shown in Fig. 5c. With a better fit to the observed OSL yield data and more detailed knowledge of its time dependence, the correction could be further improved.

### Acute versus chronic irradiation

**Fig. 8:**
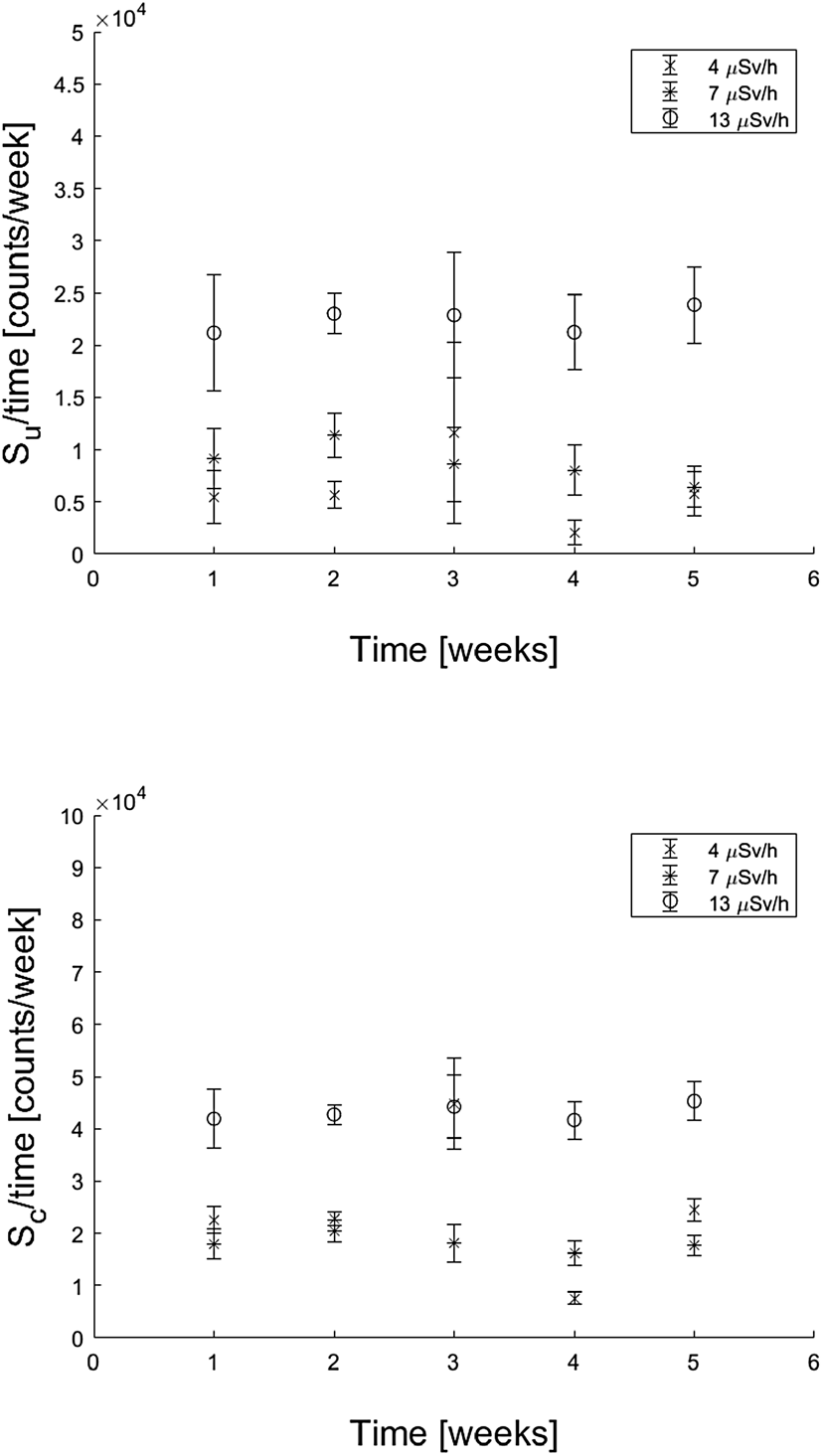
(Top) *S*_*u*_*/*time and (bottom) *S*_*c*_/time of five NaCl pellets in free air, obtained at three different dose rates from a ^137^Cs source. Five pellets for each dose rate were read once per week for 5 weeks. The reference dose rates for the different data series are given as ambient dose equivalent as measured by a handheld detector

**Fig. 9:**
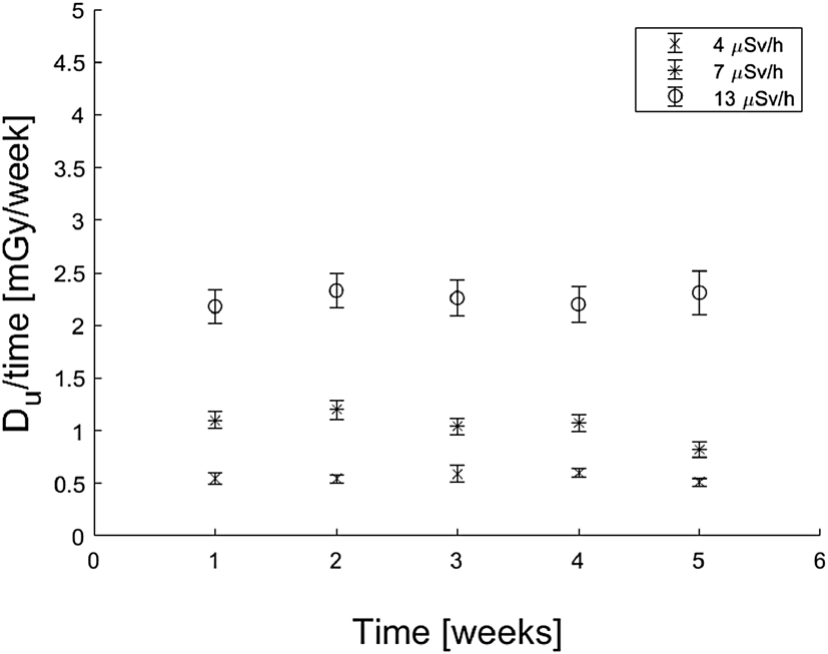
Weekly absorbed dose, *D*_*u*_*/*time, in NaCl pellets at three different dose rates as calculated by Eq. 1 from the signals in Fig. 8. The reference dose rates for the different data series are given as ambient dose equivalent as measured by a handheld detector

For the extended irradiations, the average *S*_*u*_/time (Fig. 8 top) and *S*_*c*_/time (Fig. 8 bottom) appear to be stable over the investigated 5-week period for all three dose rates. There are some values that deviate from the overall trend, which may be attributed to pellets being broken or to any variation in the amount of NaCl in each pellet. For some measurement points, the data deviate from the overall trend for both *S*_*u*_ and *S*_*c*_. This supports the possibility of a varying amount of NaCl in each pellet being the reason, as this is cancelled out in the *D*_*u*_ calculations (Fig. 9). The results in Fig. 8 can be compared to those of the acute irradiations shown in Fig. 5a, b. In Fig. 5b, there is a clear decrease in *S*_*c*_ with time, while this effect is not seen for *S*_*c*_ in Fig. 8, even though both Figs. 5b and 8 show the *S*_*c*_ values obtained after a calibration dose administered by the ^90^Sr/^90^Y source at different times up to at least a month. The estimated *D*_*u*_/time remains stable as a function of the measured time interval (Fig. 9). Unlike the situation with acute irradiations (Fig. 5c), there is no observed inverse fading for prolonged irradiations. This may be the result of a continuous fading and stabilisation of the signal over time for extended irradiations.

Additional extended exposure experiments are needed to investigate if the decrease in signal efficiency of the NaCl pellets can also be seen in other exposure situations, for example, in environmental monitoring. If not, there is no need for a correction of extended measurements.

## Conclusions

In this study, the OSL signal in NaCl pellets (0.8 mm thick and 4 mm in diameter) has been investigated for its potential use in prospective dosimetry using blue light continuous wave OSL. Of specific importance for dosimetry using such pellets outside the laboratory are the timing between production and irradiation and the timing between irradiation and subsequent readout protocol, including optimised preheat. The results indicate the following:

- A preheat at 100 ºC or a pause of 3600 s between irradiation and readout gives a reproducible OSL signal.
- Taking into account the measurement uncertainties, the OSL signal from the NaCl pellets appears to be stable over time, at least over 4 weeks.
- To more accurately estimate absorbed doses, a correction may be considered for the decrease in OSL signal yield with time.
- The change in OSL signal yield with time can be corrected if the rate of change in a particular salt brand is known beforehand.
- If acute irradiation is closely followed by the readout, the OSL signal yield does not need to be corrected.
- For chronic exposures there is no observable change in OSL signal yield for up to at least 5 weeks of exposure, indicating possible stabilisation of traps with time.
- The results obtained in this work can be applied not only for prospective but also for retrospective dosimetry.

The results of this study show that the NaCl pellets can be used for long-term measurements which is essential for personal dosimetry. Using NaCl pellets for prospective dosimetry is a promising, cost-effective, and accessible complement to commercially available alternatives for accurate absorbed dose determinations.

Future work includes investigations of the thermoluminescence signal from different types of NaCl to further optimise the preheat temperature and to explain some of the phenomena addressed in this work, especially in terms of signal response for long-term exposures.

## References

[CR1] Ademola JA (2017). Luminescence properties of common salt (NaCl) available in Nigeria for use as accident dosimeter in radiological emergency situation. J Radiat Res Appl Sci.

[CR2] Bailey RM (2000). Interpretation of quartz optically stimulated luminescence equivalent dose versus time plots. Radiat Meas.

[CR3] Bailey RM, Adamiec G, Rhodes EJ (2000). OSL properties of NaCl relative to dating and dosimetry. Radiat Meas.

[CR4] Berger MJ, Coursey JS, Zucker MA, Chang J (2005), ESTAR, PSTAR, and ASTAR: computer programs for calculating stopping-power and range tables for Electrons, Protons, and Helium Ions (version 1.2.3). [Online] Available: https://physics.nist.gov/Star [2020, August 20]. National Institute of Standards and Technology, Gaithersburg, MD

[CR5] Bernhardsson C, Christiansson M, Mattsson S, Rääf CL (2009). Household salt as a retrospective dosemeter using optically stimulated luminescence. Radiat Environ Biophys.

[CR6] Bernhardsson C, Matskevich S, Mattsson S, Rääf C (2012). Comparative measurements of the external radiation exposure in a 137Cs contaminated village in Belarus based on optically stimulated luminescence in NaCl and thermoluminescence in LiF. Health Phys.

[CR7] Biernacka M, Majgier R, Maternicki K, Liang M, Mandowski A (2016). Peculiarities of optically stimulated luminescence in halite. Radiat Meas.

[CR8] Christiansson M, Bernhardsson C, Mattsson S, Rääf CL (2011). Using an optimised OSL single-aliquot regenerative dose protocol for low-dose retrospective dosimetry on household salt. Radiat Prot Dosimetry.

[CR9] Christiansson M, Bernhardsson C, Geber-Bergström T, Mattsson S, Rääf C (2014). Household salt for retrospective dose assessments using OSL: signal integrity and its dependence on containment, sample collection, and signal readout. Radiat Environ Biophys.

[CR10] Christiansson M, Bernhardsson C, Geber-Bergstrand T, Mattsson S, Rääf C (2018). OSL in NaCl vs. TL in LiF for absorbed dose measurements and radiation quality assessment in the photon energy range 20 keV to 1.3 MeV. Radiat Meas.

[CR11] Ekendahl D, Bulánek B, Judas L (2016). A low-cost personal dosemeter based on optically stimulated luminescence (OSL) of common household salt (NaCl). Radiat Meas.

[CR12] Elashmawy M (2018). Study of constraints in using household NaCl salt for retrospective dosimetry. Nucl Instruments Methods Phys Res Sect B Beam Interact Mater Atoms.

[CR13] Hunter PG, Spooner NA, Smith BW, Creighton DF (2012). Investigation of emission spectra, dose response and stability of luminescence from NaCl. Radiat Meas.

[CR14] Majgier R, Rääf CL, Mandowski A, Bernhardsson C (2019). OSL properties in various forms of KCl and NaCl samples after exposure to ionizing radiation. Radiat Prot Dosim.

[CR15] McKeever SWS, Moscovitch M (2003). On the advantages and disadvantages of optically and thermoluminescence dosimetry. Radiat Prot Dosim.

[CR16] Spooner NA, Smith BW, Williams OM, Creighton DF, McCulloch I, Hunter PG, Questiaux DG, Prescott JR (2011). Analysis of luminescence from common salt (NaCl) for application to retrospective dosimetry. Radiat Meas.

[CR17] Thomsen KJ (2004). Optically stimulated luminescence techniques in retrospective dosimetry using single grains of quartz extracted from unheated materials, Doctoral dissertation.

[CR18] Waldner L (2017) NaCl pellets for improved dosimetry. Master’s theses. Lund University, Malmö

[CR19] Waldner L, Bernhardsson C (2018). Physical and dosimetric properties of NaCl pellets made in-house for the use in prospective optically stimulated luminescence dosimetry applications. Radiat Meas.

